# Cytokine and Chemokine Profiling in Cats With Sepsis and Septic Shock

**DOI:** 10.3389/fvets.2020.00305

**Published:** 2020-05-29

**Authors:** Roberta Troia, Giulia Mascalzoni, Chiara Agnoli, Denise Lalonde-Paul, Massimo Giunti, Robert Goggs

**Affiliations:** ^1^Department of Veterinary Medical Sciences, Alma Mater Studiorum, University of Bologna, Bologna, Italy; ^2^Department of Clinical Sciences, College of Veterinary Medicine, Cornell University, Ithaca, NY, United States

**Keywords:** feline, interleukin, IL-6, IL-8, KC-Like, RANTES, multiplex

## Abstract

**Background:** Sepsis is a life-threatening condition associated with an exacerbated production of both pro- and anti-inflammatory cytokines that can promote a hyperactive response to infection or induce immunoparalysis. Data regarding the immune response to sepsis in cats are scarce. Establishing the profiles of cytokines and chemokines in feline sepsis to characterize the nature of the immune responses to sepsis might enable individualized treatments to be developed and targeted.

**Objective:** To evaluate the cytokine and chemokine network in cats with sepsis and septic shock, and to investigate the associations of these analytes with disease severity and outcome.

**Methods:** Blood samples prospectively collected at presentation of cats with sepsis and septic shock to two veterinary teaching hospitals were analyzed. Forty healthy cats were included as controls. A 19-plex feline cytokine/chemokine magnetic bead assay system was used to measure analytes in citrated plasma samples. Cytokine concentrations were compared between groups using the Kruskal-Wallis test with Dunn's *post-hoc* correction for multiple comparisons. Cytokine concentrations were compared between survivors and non-survivors with the Mann-Whitney U test. Odds ratios were calculated using logistic regression. A multivariable logistic regression model for prediction of septic shock was constructed.

**Results:** The study enrolled 35 septic cats. Many cytokines were undetectable in both sick and healthy control cats and were excluded from subsequent analyses. Comparisons of cytokine concentrations among healthy controls, cats with sepsis (*n* = 12) and cats with septic shock (*n* = 23) revealed that sick cats (sepsis or septic shock) had significantly higher plasma concentrations of IL-6, IL-8, KC-like, and RANTES compared to healthy controls. The combination of MCP-1, Flt-3L, and IL-12 was predictive of septic shock. None of the cytokines analyzed was predictive of outcome in this study population.

**Conclusion:** Plasma concentrations of IL-6, IL-8, KC-like, and RANTES are increased in cats with sepsis and may play important roles in pathogenesis. Multivariable modeling suggested that analysis of cytokines might aid differentiation of septic shock from sepsis. None of the cytokines analyzed was predictive of outcome. Measurement of these cytokines might enable future studies to better diagnose and characterize feline sepsis and septic shock.

## Introduction

The inflammatory response in sepsis involves leukocyte activation through the recognition of pathogen and damage associated molecular patterns by Toll-like receptors and the subsequent production of pro- and anti-inflammatory cytokines (1). These small pleotropic proteins promote and orchestrate a variety of inflammatory reactions and cell-cell signaling events and enhance pathogen elimination (2). Early descriptions of sepsis pathophysiology emphasized an excessive pro-inflammatory response (3, 4). This understanding underpinned the concept of the systemic inflammatory response syndrome (SIRS) that was the basis for the 2001 definition of sepsis (5). Improvements in understanding of sepsis pathophysiology drove a recent redefinition in human medicine with sepsis now described as the life-threatening organ dysfunction caused by a dysregulated host response to infection (6). This new definition encompasses a more nuanced understanding of the immune responses to infection.

Recently the importance of sepsis-induced immunoparalysis in the morbidity and mortality of sepsis has been recognized (7). In human medicine, there is huge potential for precision medicine through immunophenotyping to provide targeted interventions specific to the patient's underlying pathology. For instance, those patients with a hyperactive immune response may benefit from anti-inflammatory therapies, while those with suppressed and inadequate immune responses may benefit from immunostimulatory therapies. Achieving a better understanding of the nature of the inflammatory and immune responses to sepsis is a crucial first step in this endeavor in veterinary medicine. Leukocyte activity and cytokine release are profoundly impaired during immunoparalysis in which immune cell apoptosis is a central phenomenon (8). Both hyper- and hypo-inflammation can be present in different subsets of septic patients, explaining the overall failure of undifferentiated immunomodulatory therapy for sepsis following a “one-size fits all” approach (9). Accordingly, profiling cytokines and chemokines may provide a snapshot of the current immunological state of a septic cat that could be used to tailor treatment (8, 9).

Immunophenotyping data characterizing sepsis are scarce in veterinary medicine and are predominantly available for dogs (10–13). Recognizing and characterizing sepsis in cats is more challenging than in dogs (14, 15). This is likely due to the lack of extensive validation of feline SIRS criteria in clinical studies, the limited diagnostic value of leukocyte counts in cats, and the unique manifestations of sepsis in this species (16, 17). Toxic neutrophil changes, serum amyloid-A (SAA), and selected cytokines including TNF and IL-6 have been described as potential diagnostic and prognostic biomarkers in cats with sepsis (16–18), but independent evaluation of these biomarkers in a large population of septic cats is currently lacking.

Recently, the advent of multiplex technology has increased the ability to screen for multiple biomarkers of systemic conditions. Multiplex assays are able to simultaneously and rapidly measure a wide variety of immunologically active proteins in small volumes of biological samples. Feline-specific multiplex assays have recently been used to measure cytokines in serum and plasma samples from cats affected by heterogeneous diseases, idiopathic cystitis and degenerative joint disease (19–22), and demonstrate reliable cytokine detection when the blood concentration of the analytes is high.

The evaluation of cytokines and chemokines profiles in cats with sepsis and with septic shock may offer novel insights into the immune pathways of this syndrome and identify potential non-invasive biomarkers to diagnose, prognosticate and predict the therapeutic response of the disease. The aims of the current study were to describe the cytokine and chemokine network in cats with sepsis and septic shock using a commercially available multiplex magnetic bead assay and to investigate the associations of these analytes with disease severity and outcome. It was hypothesized that concentrations of pro- and anti-inflammatory cytokines in cats with sepsis and septic shock would differ from those of healthy cats, and that these concentrations would correlate with sepsis severity and prognosis.

## Materials and Methods

### Study Setting and Population

Data for the current analysis are a subset of the data collected for studies on the characterization of sepsis and septic shock in cats prospectively enrolled between 2016 and 2019 at the veterinary teaching hospitals at the University of Bologna, Italy and Cornell University, Ithaca NY. These studies were approved by the respective local Institutional Animal Care and Use Committees. Respective primary clinicians determined all aspects of patient management. The study population of 35 cats (26 Bologna, 9 Cornell) represents 17% (9/54) of the cats with sepsis hospitalized at Cornell University during the study period and 15% (26/176) of the cats with SIRS or sepsis hospitalized at the University of Bologna during the study period.

Cats were diagnosed with sepsis if at least 2/4 SIRS criteria were satisfied (15), and if an infection was confirmed by means of cytology, microbiology, histopathology or real-time polymerase chain reaction. Cats with sepsis were diagnosed with septic shock if they had persistent hyperlactatemia (lactate >2 mmol/L for >12 h despite fluid resuscitation or clinical euvolemia) and/or hypotension (systolic blood pressure <90 mmHg) requiring vasopressor support despite adequate fluid resuscitation (23). Cats were eligible for inclusion if they were hospitalized in the intensive care unit (ICU) for at least 12 h, and if an aliquot of citrated plasma collected upon admission and stored frozen at −80°C was available for analysis.

Forty healthy privately-owned cats were enrolled as controls with informed owner consent. These cats were eligible if they had no history or evidence of recent or chronic medical conditions, and were classified as healthy based on history, physical examination, complete blood count and serum chemistry results.

### Data Collection

For cats with sepsis, the following parameters were recorded at study enrollment: medical history including comorbidities, previous treatments and current therapies and physical examination findings including non-invasive systolic blood pressure measurement (petMAP Graphic, Ramsey Medical, Sydney, Australia; Minidop ES-100 VX, Hadeco, Kawasaki, Japan; Cardell 9401, Midmark, Dayton, OH). Blood gas and electrolyte analyses and blood lactate measurement were performed using point-of-care analyzers (ABL800 FLEX, Radiometer Medical ApS, Denmark; RapidPoint 500, Siemens Medical Solutions, Norwood, MA). Complete blood count and serum chemistry analyses including serum amyloid-A (SAA) evaluation were performed using automated analyzers (ADVIA 2120, Siemens Healthcare Diagnostics, Tarrytown, NY; Olympus AU 400, Olympus/Beckman Coulter, Brea, CA; Cobas, Roche, Indianapolis, IN). Patient data were used to calculate the feline Acute Patient Physiologic and Laboratory Evaluation (APPLE_full_ and APPLE_fast_) scores as previously described (24). Outcome was defined as survival to hospital discharge, death or euthanasia for disease severity. Cases euthanized for financial reasons were excluded from outcome calculations.

### Feline Cytokine Assays

For cytokine measurements, citrated plasma was generated by centrifugation of whole blood for 10 min at 1,370 g. After centrifugation, plasma was frozen at −80°C until cytokine analyses were performed. An antibody-coated microsphere-based multiplex feline-specific cytokine immunoassay kit (Milliplex MAP Feline Cytokine/Chemokine Magnetic Bead Panel, Millipore-Sigma, Billerica, MA) was used to measure 19 cytokines as previously described (19–22). Analytes included in the kit were Fas, FMS-like tyrosine kinase-3 ligand (Flt-3L), granulocyte macrophage-colony stimulating factor (GM-CSF), interferon-γ (IFN-γ), interleukin 1beta (IL-1β), IL-2, platelet-derived growth factor-BB (PDGF-BB), IL-12, IL-13, IL-4, IL-6, IL-8, keratinocyte chemoattractant-like (KC-like), stromal cell-derived factor 1 (SDF-1), Regulated upon Activation, Normal T cell Expressed, and Secreted (RANTES), stem cell factor (SCF), C-C motif chemokine ligand-2 (CCL-2) [also known as monocyte chemoattractant protein-1 (MCP-1)], tumor-necrosis factor-α (TNF-α), and IL-18. Kits were used according to manufacturer recommendations. Cytokine concentrations were measured in triplicate and the mean values used for subsequent analyses. Quality controls were run, in duplicate on each plate. Two different plates analyzed on different days were necessary to analyze all of the samples included in the present study. The mean fluorescence intensity (MFI) values could not therefore be directly compared and are not reported (25). Hence, results of each analyte for each sample were expressed as observed concentrations, and calculated using a standard curve generated from the standards and blank provided by the manufacturer. Where cytokine concentrations were recorded as below the measurable range of the assay or lower limit of detection (LLD) the manufacturer's stated minimum detectable concentrations (pg/mL) were imputed as follows to facilitate statistical analyses: 4.0 (Fas), 4.0 (Flt-3L), 6.0 (GM-CSF), 43.0 (IFN-γ), 14.0 (IL-1β), 4.0 (IL-2), 198.0 (PDGF-BB), 9.0 (IL-12), 7.9 (IL-13), 30.0 (IL-4), 25.0 (IL-6), 7.0 (IL-8), 1.0 (KC), 97.0 (SDF-1), 1.0 (RANTES), 48.0 (SCF), 164.0 (MCP-1), 7.0 (TNF-α), 30.0 (IL-18). Plates were analyzed using a dedicated plate reader and software (Bio-Plex System with Luminex xMap Technology, Bio-Rad, Hercules, CA).

### Statistical Analyses

Prior to test selection, data were assessed for normality with the D'Agostino Pearson test. Descriptive statistics were calculated and expressed as median (min-max) or mean ± standard deviation. Non-parametric statistics (Mann-Whitney-*U* test, Kruskal Wallis test with Dunn's correction for multiple comparisons) were used to compare cytokines between different groups (survivors vs. non-survivors; controls vs. sepsis vs. septic shock). Logistic regression and construction of receiver operating characteristic (ROC) curves was performed to calculate odds ratios and confidence intervals for prediction of disease status. Multivariable logistic regression was performed to identify combinations of cytokines that could differentiate septic shock from sepsis. Potential predictor variables were chosen based on univariate analyses. All potential predictors were simultaneously entered into the model to maximize predictive ability. Classification tables were used to assess model accuracy. Calibration of the final model was determined using the Hosmer–Lemeshow goodness-of-fit (model rejected if *P* < 0.05) and model utility assessed by calculation of the Nagelkerke *R*^2^ value. Multicollinearity was assessed by calculation of the variation inflation factor (VIF), with values <2.5 determined to be acceptable. The ability of the final model to discriminate the outcome was determined by calculating area under receiver-operating characteristic curves (AUROC). Statistical analyses were performed with commercial software (Prism 8.4.1, GraphPad, La Jolla, CA; SPSS 26, IBM, Armonk, NY). Alpha was set at *P* < 0.05.

## Results

### Demographic Data

The study enrolled 35 septic cats and 40 controls. Among the septic cats, there were 33 domestic shorthaired cats, 1 Bengal and 1 Siamese. There were 12 male neutered cats, 5 male intact cats, 10 spayed female cats, 8 intact female cats. The median age was 3 years (0.3–16), and the median body weight was 3.9 kg (0.7–7.3). The median number of SIRS criteria identified upon admission was 3 (2–4); 11/35 cases satisfied only 2/4 of the SIRS criteria, while 24/35 satisfied ≥3/4 SIRS criteria. The median duration of hospital stay was 4 days (0.5–18). Twelve cats out of 35 had sepsis, while 23/35 cats had septic shock. Among the latter, 6/23 cats had persistent hyperlactatemia (>12 h) despite fluid resuscitation, while 17/23 had persistent hypotension and required vasopressor support. Underlying causes for sepsis in the overall study population included pyothorax (*n* = 7), septic peritonitis (*n* = 7), bite wounds (*n* = 6), feline panleukopenia (*n* = 4), pyelonephritis (*n* = 4), pyometra (*n* = 4), bacterial cholangitis (*n* = 2), abdominal abscess (*n* = 1). Twenty-one cats survived to hospital discharge, while 14/35 cats died or were euthanized, equivalent to an overall case fatality rate of 40%. Descriptive statistics for selected clinical and clinicopathological variables in the study population are summarized in Table 1.

**Table 1:** Results of descriptive statistics for selected clinical and clinicopathological variables in cats with sepsis and septic shock.

**Variable**	**Controls (*n* = 40)**	**Sepsis (*n* = 12)**	**Septic shock (*n* = 23)**	**Statistical test used**
T (°C)	NA	38.6 ± 1.5^a^	36.7 ± 2.6^b^	*t*-test
Heart rate (bpm)	NA	186 ± 41^a^	168 ± 39^a^	*t*-test
Respiratory rate (rpm)	NA	45 (24–120)^a^	38 (20–100)^a^	M-W
SBP (mmHg)	NA	129 ± 25^a^	98 ± 20^b^	*t*-test
APPLE full score	NA	37 ± 8.5^a^	48 ± 10^b^	M-W
APPLE fast score	NA	22 (8–17)^a^	31 ± 10^b^	M-W
n. SIRS criteria	NA	2 (2–4)^a^	3 (1–4)^b^	M-W
**HEMATOLOGY**				
Hb (g%)	14.0 (12.4–16.9)^a^	11.2 ± 1.9^b^	9.9 ± 3.2^b^	K-W
HCT (%)	42.0 (36.0–50.0)^a^	34.1 ± 5.9^b^	29.7 ± 8.6^b^	K-W
MCV (fL)	44.7 ± 3.7^a^	44.9 ± 5.0^a, b^	41.9 ± 2.9^b^	ANOVA
MCHC (g%)	33.7 ± 0.7^a^	32.7 ± 1.3^a^	33.0 ± 1.8^a^	ANOVA
RDW (%)	14.8 (13.9–17.5)^a^	15.6 ± 1.6^a, b^	16.0 ± 1.5^b^	K-W
WBC (×10^9^/L)	9.15 ± 2.4^a^	13.4 ± 9.8^a, b^	17.6 ± 18.7^b^	ANOVA
Lymphocytes (×10^9^/L)	2.95 (1.2–6.6)^a^	1.5 (0.2–7.0)^b^	1.1 (0.0–2.6)^b^	K-W
Neutrophils (×10^9^/L)	4.55 (2.1–10.9)^a^	9.5 ± 9.5^a^	14.2 ± 16.1^a^	K-W
Monocytes (×10^9^/L)	0.19 ± 0.11^a^	0.25 (0.0–3.9)^a^	0.3 (0.0–3.3)^a^	K-W
Eosinophils (×10^9^/L)	0.6 ± 0.4^a^	0.08 (0.0–0.8)^b^	0.06 (0.0–1.6)^b^	K-W
MPV (fL)	16.6 ± 4.3^a^	15.9 ± 6.1^a^	17.9 ± 0.7^a^	ANOVA
Platelets (×10^9^/L)	303 ± 72.6^a^	219 ± 105.6^a^	222 ± 164.3^a^	ANOVA
**CHEMISTRY**				
Glucose (mg/dL)	93 (62–313)^a^	175 ± 35^b^	149 (22–607)^b^	K-W
Creatinine (mg/dL)	1.15 ± 0.27^a^	1.05 (0.40–23.80)^a^	1.57 (0.46–19.47)^a^	K-W
Urea (mg/dL)	24.5 (16.0–39.0)^a^	36.3 ± 42.3^a^	72.7 (24.0–868.0)^b^	K-W
Phosphorus (mg/dL)	4.3 ± 0.9^a^	4.3 (2.6–20.1)^a, b^	6.3 (2.0–33.7)^b^	K-W
Total proteins (mg/dL)	7.21 ± 0.56^a^	6.39 ± 1.29^b^	6.03 ± 1.07^b^	ANOVA
Albumin (mg/dL)	3.60 ± 0.30^a^	2.82 ± 0.63^b^	2.32 ± 0.54^c^	ANOVA
A/G	1.03 ± 0.19^a^	0.81 ± 0.18^b^	0.64 ± 0.18^c^	ANOVA
AST (U/L)	25.0 (13.0–58.0)^a^	68.5 ± 45.4^b^	108.5 (20.0–2725)^b^	K-W
ALT (U/L)	50.5 (29.0–101.0)^a^	49.5 (27.0–133.0)^a^	55.0 (4.0–1284)^a^	K-W
GGT (U/L)	0.0 (0.0–3.0)^a^	1.3 ± 1.3^a^	0.7 ± 0.7^a^	ANOVA
ALP (U/L)	32.0 (13.0–66.0)^a^	10.5 (3.0–45.0)^b^	17.0 (1.0–307.0)^a, b^	K-W
Total bilirubin (mg/dL)	0.0 (0.0–0.1)^a^	0.18 (0.0–1.4)^b^	0.94 (0.1–7.4)^b^	K-W
Total calcium (mg/dL)	10.0 ± 0.5^a^	8.8 ± 0.85^b^	8.5 ± 1.2^b^	ANOVA
Chloride (mmol/L)	116 ± 2^a^	109 ± 6.4^b^	111 ± 11^b^	ANOVA
Sodium (mmol/L)	152 (149–165)^a^	149 (128–154)^b^	148 ± 9.6^b^	K-W
Potassium (mmol/L)	4.1 ± 0.4^a^	3.9 (3.0–10.0)^a^	4.7 (2.6–8.8)^a^	K-W
Magnesium (mg/dL)	1.82 ± 0.14^a^	2.64 ± 0.74^b^	3.2 ± 0.8^c^	ANOVA
SAA (mg/dL)	NA	117 ± 67^a^	158 ± 94^a^	*t*-test
**BLOOD GAS**				
pH		7.30 (6.78–7.39)^a^	7.21 ± 0.14^a^	M-W
HCO_3_ (mmol/L)	NA	19.2 (2.5–23.5)^a^	15.6 ± 4.9^a^	M-W
BE (mmol/L)	NA	−5.4 (−3.2 to −1.7)^a^	−11 ± 6.7^a^	M-W
Anion gap (mmol/L)	NA	25.0 (7.4–50.0)^a^	15.8 ± 6.8^b^	M-W
Ionized calcium (mmol/L)	NA	1.19 ± 0.08^a^	1.20 ± 0.12^a^	*t*-test
Lactate (mmol/L)	NA	2.2 ± 1.6^a^	3.9 (1.2–26.0)^b^	M-W

*Data are reported as mean ± SD or median (min-max) based on their distribution. Concentrations sharing a superscript letter code annotation were not significantly different, while those not sharing a letter code were significantly different*.

*A/G, albumin to globulin ratio; ALP, alkaline phosphatase; ALT, alanine transaminase; ANOVA, analysis of variance; APPLE, acute patient physiologic and laboratory evaluation; AST, aspartate transaminase; BE, base excess; GGT, ɤ- glutamyltransferase; Hb, hemoglobin concentration; HCT, hematocrit value; K-W, Kruskal-Wallis test; MCHC, mean cell hemoglobin concentration; MCV, mean corpuscular volume; MPV, mean platelet volume; RI, reference interval; M-W, Mann-Whitney U test; RDW, red cell distribution width; SAA, serum-amyloid A; SBP, systolic blood pressure; SIRS, systemic inflammatory response syndrome; T, temperature; WBC, white blood cell. NA, not applicable/not available*.

### Cytokine Concentrations

Plasma concentrations of many analytes were below the LLD for most healthy feline control samples. For the purposes of numerical comparison these were therefore expressed as the manufacturer stated minimum detectable concentrations in pg/mL. Five of the 19 analytes (Fas, GM-CSF, INFγ, IL-2, and SCF) were undetectable in all samples (healthy and diseased) and were excluded from subsequent analyses.

**Figure 1:**
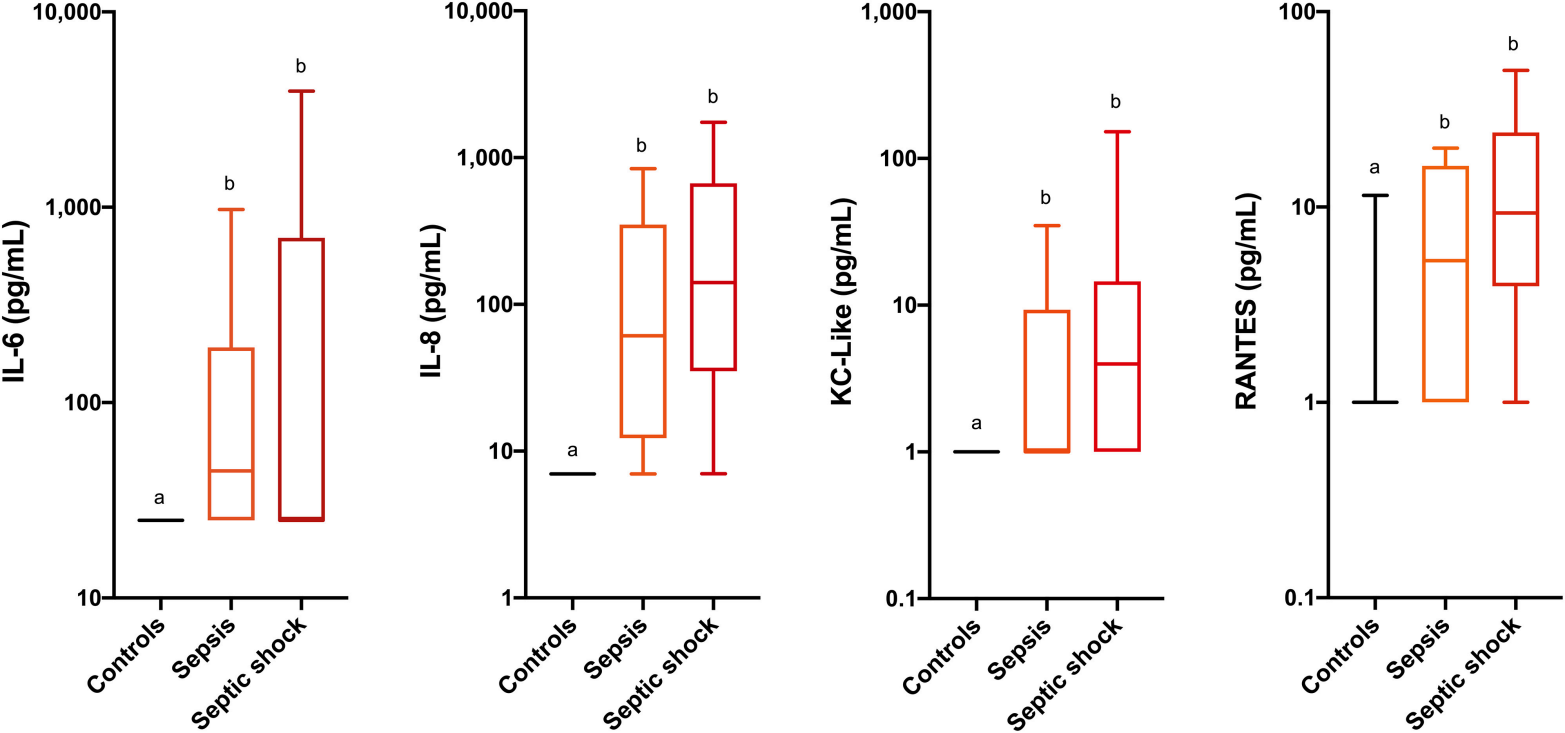
Box and whisker plots for the concentrations of interleukin-6 (IL-6), interleukin-8 (IL-8), keratinocyte-chemoattractant-like (KC-Like) and Regulated upon Activation, Normal T cell Expressed, and Secreted (RANTES) in healthy cats, cats with sepsis, and cats with septic shock. Concentrations sharing a letter code annotation were not significantly different, while those not sharing a letter code were significantly different. For cytokines with measured concentrations below those covered by the standard curve, values for the assay lower limit of detection were imputed. Hence the figures appear truncated due to imputation of the lower limits of detection. Note the logarithmic scales of the Y-axes.

**Figure 2:**
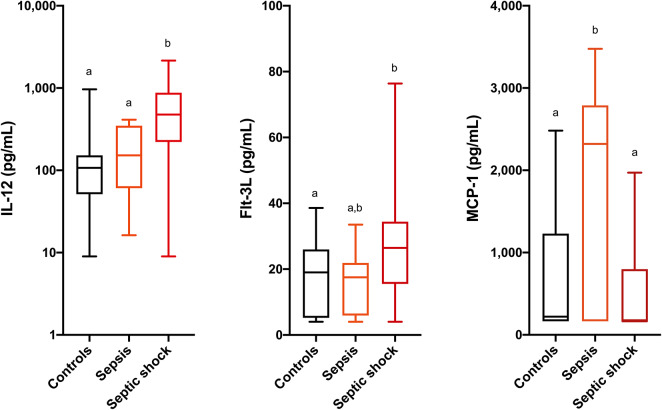
Box and whisker plots for the concentrations of interleukin-12 (IL-12), FMS-like tyrosine kinase-3 ligand (Flt-3L) and monocyte chemoattractant protein-1 (MCP-1) in healthy cats, cats with sepsis, and cats with septic shock. Concentrations sharing a letter code annotation were not significantly different, while those not sharing a letter code were significantly different. For cytokines with measured concentrations below those covered by the standard curve, values for the assay lower limit of detection were imputed. Hence the figures appear truncated due to imputation of the lower limits of detection. Note that the Y-axis scale for IL-12 is logarithmic, while the Y-axis scales for Flt-3L and MCP-1 are linear.

Comparisons of analyte concentrations among healthy controls, cats with sepsis and cats with septic shock revealed that sick cats (both with sepsis and septic shock) had significantly higher plasma concentrations of IL-6, IL-8, KC-like, and RANTES compared to healthy controls (Figure 1). No other significant differences were identified between healthy and cats with sepsis. Cats with sepsis showed significantly higher concentrations of IL-13, MCP-1, and IL-18 compared to cats with septic shock and healthy controls (Table 2). Cats with septic shock had significantly greater IL-12 concentrations than cats with sepsis and healthy controls, and greater Flt-3L concentrations compared to cats with sepsis (Figure 2).

**Table 2:** Comparison of plasma concentration of cytokines and chemokines healthy controls, cats with sepsis and cats with septic shock.

**Variable (pg/mL)**	**Controls (*n* = 40)**	**Sepsis (*n* = 12)**	**Septic shock (*n* = 23)**	**Overall *P*-value**
Flt-3L	19.01(4.0–38.6)^a^	17.5 (4.0–33.5)^a^	26.4 (4.0–76.4)^b^	0.01
IL-1β	14.0 (14.0–61.7)^a^	14.0 (14.0–53.1)^a^	14.0 (14.0–80.6)^a^	0.51
PDGF-BB	198.0 (198.0–297.4)^a^	198.0 (198.0–198.0)^a^	198.0 (198.0–1442.0)^a^	0.12
IL-12	107.1 (9.0–965.8)^a^	152.0 (16.3–412.9)^a^	476.7 (9.0–2161.0)^b^	<0.001
IL-13	7.0 (7.0–7.0)^a^	7.0 (7.0–50.3)^a^	7.0 (7.0–7.0)^b^	0.0003
IL-4	30.0 (30.0–363.7)^a^	30.0 (30.0–110.5)^a^	30.0 (30.0–95.0)^a^	0.44
IL-6	25.0 (25.0–25.0)^a^	44.7 (25.0–973.5)^b^	25.0 (25.0–3931.0)^b^	<0.001
IL-8	7.0 (7.0–7.0)^a^	61.0 (7.0–840.0)^b^	140.4 (7.0–666.0)^b^	<0.001
KC-like	1.0 (1.0–1.0)^a^	1.0 (1.0–34.8)^b^	3.9 (1.0–151.9)^b^	<0.001
SDF-1	945.7 (248.0–2233.0)^a^	598.3 (237.8–1316.0)^a^	980.7 (291.8–3566.0)^a^	0.10
RANTES	1.0 (1.0–11.46)^a^	5.3 (1.0–20.0)^b^	9.3 (1.0–50.0)^b^	<0.001
MCP-1	220.6 (164.0–2482)^a^	2320.0 (164.0–3476.0)^b^	164.0 (164.0–1971.0)^a^	0.016
TNF-α	7.0 (7.0–43.3)^a^	7.0 (7.0–26.4)^a^	7.0 (7.0–24.5)^a^	0.28
IL-18	30.0 (30.0–30.0)^a^	303.8 (30.0–1378.0)^b^	30.0 (30.0–30.0)^a^	<0.001

*Data are reported as median (min-max)*.

*Flt-3L, FMS-like tyrosine kinase-3 ligand; IL, interleukin, KC-like, keratinocyte chemoattractant-like; MCP-1, monocyte chemoattractant protein-1; PDGF-BB, platelet-derived growth factor-BB; RANTES, Regulated upon Activation, Normal T cell Expressed and Secreted; SDF-1, stromal cell-derived factor 1; TNF-α, tumor-necrosis factor-α. Concentrations sharing a letter code annotation were not significantly different, while those not sharing a letter code were significantly different*.

**Figure 3:**
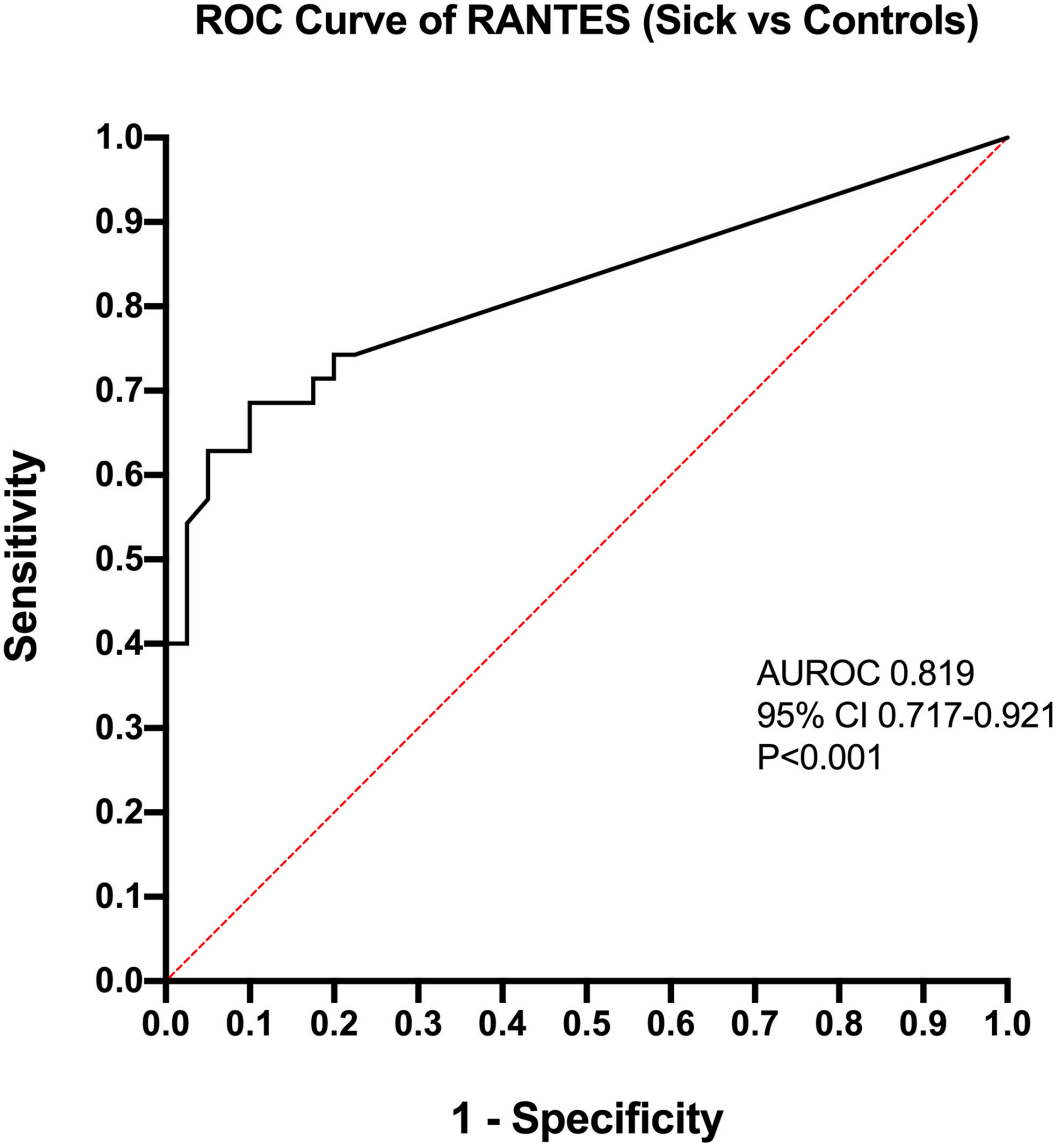
Receiver operating characteristic (ROC) curve for the differentiation of cats with sepsis (sepsis and septic shock) from healthy controls by measurement of Regulated upon Activation, Normal T cell Expressed, and Secreted (RANTES). AUROC, area under the ROC curve; CI, confidence interval.

**Figure 4:**
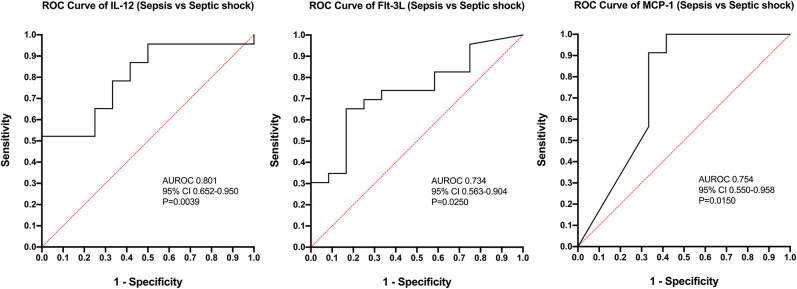
Receiver operating characteristic (ROC) curves for differentiation of cats with septic shock from those with sepsis using measurement of the concentrations of Interleukin-12, FMS-like tyrosine kinase-3 ligand, and MCP-1 monocyte chemoattractant-1. AUROC, area under the ROC curve; CI, confidence interval.

**Figure 5:**
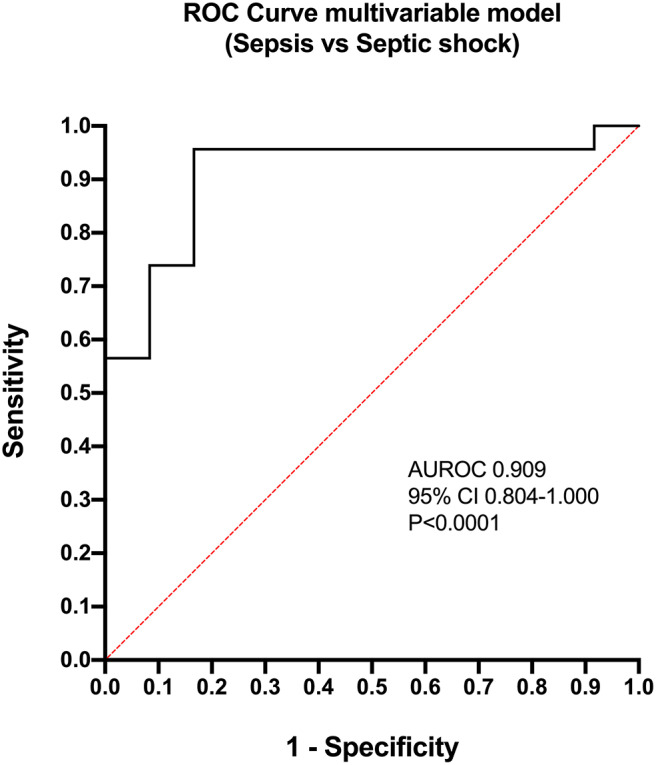
Receiver operating characteristic (ROC) curve for the multivariable model constructed for the differentiation of cats with septic shock from those with sepsis. The model contained three variables: FMS-like tyrosine kinase-3 ligand, Interleukin-12 and MCP-1 monocyte chemoattractant-1. AUROC, area under the ROC curve; CI, confidence interval.

Calculation of odds ratios (Table 3) and construction of ROC curves (Figure 3) suggested that RANTES was discriminating for the presence of sepsis (including septic shock) compared to healthy controls. Similarly, calculation of odds ratios (Table 4) and construction of ROC curves (Figure 4) suggested that IL-12, Flt-3L, and MCP-1 were capable of discriminating septic shock from sepsis. A multivariable model incorporating IL-12, Flt-3L, and MCP-1 concentrations (Table 5) was highly discriminant for septic shock (Figure 5). This model correctly classified 32/35 (91.4%) cases. The Hosmer-Lemeshow statistic for this model was 8.68, *P* = 0.370, suggesting the model was well-fitted. The Nagelkerke R^2^ value was 0.612, suggesting the model explained most of the variation in the data.

**Table 3:** Odds ratios for the differentiation of sick cats (i.e., those with sepsis or septic shock) from healthy controls based on measured cytokine concentrations.

**Cytokine**	**Odds ratio**	**95% CI**	***P*-value (OR)**	**AUROC**	***P*-value (AUROC)**
IL-6*	1.427	0.000 – ∞	0.984	0.700	0.001
IL-8*	2.766	0.000 – ∞	0.976	0.929	<0.001
KC-Like*	162.058	0.000 – ∞	0.990	0.743	<0.001
RANTES	1.392	1.146 – 1.693	0.001	0.819	<0.001

Odds ratio values <1 indicate that decreases in the respective cytokine concentration decrease the likelihood of septic shock, while odds ratio values >1 indicate that increases in the respective cytokine concentration increase the likelihood of septic shock. Statistics labeled with * are biased due to ties (i.e., multiple healthy controls and patients with sepsis or septic shock had the same measured cytokine concentration). AUROC, area under the receiver operating characteristic curve; CI, confidence interval; OR, odds ratio. KC-Like, keratinocyte chemoattractant-like; IL, interleukin; RANTES, Regulated upon Activation, Normal T Cell Expressed and Secreted.

**Table 4:** Odds ratios for the differentiation of cats with sepsis from those with septic shock based on measured cytokine concentrations.

**Cytokine**	**Odds ratio**	**95% CI**	***P*-value (OR)**	**AUROC**	***P*-value (AUROC)**
IL-12	1.006	1.002 – 1.012	0.024	0.801	0.004
Flt-3L	1.073	1.011 – 1.164	0.048	0.734	0.025
MCP-1	0.999	0.998 – 1.000	0.004	0.754	0.015
IL-13*	0.248	0.000 – ∞	0.999	0.625	0.242
IL-18*	0.904	0.000 – ∞	0.904	0.833	<0.001

Odds ratio (OR) values <1 indicate that decreases in the respective cytokine concentration decrease the likelihood of septic shock, while OR >1 indicate that increases in the respective cytokine concentration increase the likelihood of septic shock. Statistics labeled with * are biased due to ties (i.e., multiple patients with septic shock and with sepsis had the same measured cytokine concentration). AUROC, area under the receiver operating characteristic curve; CI, confidence interval; Flt-3L, FMS-like tyrosine kinase-3 ligand; IL, interleukin; MCP-1 monocyte chemoattractant-1.

**Table 5:** Multivariable logistic regression model for differentiation of sepsis from septic shock.

**Variable**	**Model parameter estimate (ln OR)**	**SE**	***P*-value**	**OR**	**95% CI**	**VIF for other variables**	***R*^**2**^ with other variables**
Intercept [Constant]	−0.476	1.139	0.676	0.621	–	–	–
Flt-3L (pg/mL)	0.056	0.055	0.310	1.058	0.97–1.20	1.212	0.175
IL-12 (pg/mL)	0.004	0.003	0.176	1.004	1.00–1.01	1.307	0.235
MCP-1 (pg/mL)	−0.001	0.001	0.013	0.999	0.99–1.00	1.088	0.081

*Flt-3L, FMS-like tyrosine kinase-3 ligand; IL, interleukin; ln, natural logarithm; MCP-1 monocyte chemoattractant-1; OR, odds ratio; SE, standard error; VIF, variance inflation factor*.

Comparisons between survivors and non-survivors were only performed for IL-6, IL-8, KC-like, and RANTES because a significant difference between healthy and sick cats was documented only for these four analytes. No significant relationships between outcome and any of these cytokine concentrations were observed (Table 6).

**Table 6:** Comparison of plasma concentration of selected cytokines and chemokines (IL-6, IL-8, KC-like, and RANTES) in septic cats classified as survivors and non-survivors.

**Variable (pg/ml)**	**Survivors (*n* = 21)**	**Non-survivors (*n* = 14)**	***P*-value**
IL-6	25.0 (25.0–3931.0)	25.0 (25.0–2905.0)	0.609
IL-8	77.9 (7.0–1735.0)	173.7 (7.0–981.4)	0.588
KC-like	1.0 (1.0–151.9)	4.9 (1.0–34.1)	0.574
RANTES	9.3 (1.0–50.0)	5.1 (1.0–44.8)	0.148

*Data are reported as median (min-max)*.

*IL, interleukin; KC-like, keratinocyte chemoattractant-like; RANTES, Regulated upon Activation, Normal T cell Expressed and Secreted*.

## Discussion

Cytokines directly or indirectly affect every tissue in the body and play multiple roles integral to host defense against infection. The present study aimed to describe the cytokine and chemokine network in cats with sepsis and septic shock and to investigate the associations of these analytes with disease severity and outcome. Various proinflammatory cytokine concentrations were significantly different between healthy cats and those with sepsis and septic shock, but none of these cytokines was predictive of outcome in these patients. Specifically, IL-6, IL-8, KC-like, and RANTES were the biomarkers that were most discriminating for sepsis in cats.

IL-6 is a multifunctional cytokine produced by various cells, that has both pro- and anti-inflammatory activity and is involved in a multitude of immune responses. In the early phase of sepsis, IL-6 is rapidly expressed and its concentration remains increased for a prolonged period compared to other inflammatory mediators. This makes it a potentially useful biomarker of systemic inflammation (8, 18, 26). Recently, IL-6 has been highlighted as a novel diagnostic biomarker for neonatal and adult sepsis in people with a moderate diagnostic accuracy similar to procalcitonin (27–30) and in dogs with septic peritonitis (12). Additionally, increased IL-6 concentrations correlate with the risk of death in sepsis in humans and dogs (8, 31, 32). In a previous report, cats with sepsis were more likely to have detectable plasma IL-6 concentrations compared to cats with SIRS and controls, and greater IL-6 concentrations were associated with non-survival (18). In contrast, IL-6 concentrations were not prognostic in the present study.

The chemokine IL-8, also called chemokine (C-X-C motif) ligand 8 (CXCL8), is a major chemotactic factor in acute inflammation (33). It is responsible for neutrophil recruitment, margination, and tissue infiltration. IL-8 has received attention as a marker of sepsis and bacteremia in people (26, 27, 34) and it may be a useful marker in dogs with polyarthritis, sepsis and trauma (13, 35, 36). Its specificity for sepsis is not known in cats, but increased IL-8 concentrations have been documented in the course of sterile inflammatory diseases like feline idiopathic cystitis and degenerative joint disease (20, 22).

The chemokine RANTES, also known as chemokine (C-C motif) ligand 5 (CCL5), is involved in leukocyte migration. Inverse correlations have been reported between its circulating concentrations and mortality in sepsis in humans, suggesting it has a beneficial proinflammatory role in infection states (26). Keratinocyte chemoattractant-like is a chemoattractant factor that has been recently reported as a diagnostic biomarker in canine diseases (pyometra, babesiosis, sepsis) (10, 13, 37, 38). No previous data about the concentrations or functions of RANTES and KC-like are available in healthy or in sick cats.

Although IL-6, IL-8, KC-like, and RANTES acted as inflammatory mediators in our population of septic cats, they had no prognostic value, and they were not reflective of sepsis severity. Whether the lack of prognostic significance is true in feline sepsis, or it has been biased by the heterogeneity of underlying causes for sepsis and different immune status and immune phenotypes of enrolled patients, remains a question to be addressed in future studies. It is possible that the lack of correlation between cytokine concentrations and disease severity may reflect limitations of the SIRS criteria and may question the validity of feline SIRS criteria to for identification and characterization of sepsis in cats. The present study used the presence of at least 2/4 SIRS criteria to identify eligible cats for this study because they enabled swift, simple and objective identification of the potential presence of systemic inflammation. The choice to enroll cats using less stringent criteria in the present study [2/4 rather than 3/4 as proposed by Brady et al. (14)], was based on the study by Babyak and Sharp (15) who noted that the SIRS criteria were insensitive to cats with confirmed infection and the presence of organ dysfunction. Similarly, in people these criteria are neither sensitive or specific for sepsis (39, 40). As a result, the recent redefinition of sepsis in human medicine used a data-driven approach to derive novel clinical criteria for sepsis identification (23). An equivalent redefinition of sepsis in veterinary medicine had not been undertaken and hence the SIRS criteria remained the best available strategy (41). The SIRS criteria in cats have not been as thoroughly evaluated as those for dogs (42), however. It is probable that some cats did not satisfy SIRS criteria despite having severe conditions, such as pyothorax that are associated with sepsis and hence were not included in this study. Likewise, some cats that met SIRS criteria might not have genuinely had systemic inflammation. All SIRS criteria should only be applied to patients with compatible history, clinical signs, and physical examination findings that raise concern for sepsis or another disease process likely to cause a severe physiologic stress response. These various shortcomings of the SIRS criteria might have limited the strength of relationships between these parameters and the cytokine concentrations measured in the present study.

The present study defined septic shock based on the presence of persistent hyperlactatemia or the need for vasopressors to support blood pressure. The vasopressor requirement is consistent with the prevailing definitions of septic shock in veterinary and human medicine (5, 23), while the presence of persistent hyperlactatemia despite fluid resuscitation suggests ongoing hypoperfusion, inadequate tissue oxygen utilization or continued release of increased catecholamine concentrations (43). Hyperlactatemia was recently incorporated into the human sepsis 3 definition because of its association with increased in-hospital mortality (23). It should be noted that sampling technique and patient restraint might have impacted the measured lactate concentrations. It is less likely that this would have persistently increased lactate concentrations, but the possibility that occasional measured values were artifactually increased cannot be completely excluded. The significantly increased lactate, significantly lower blood pressure, greater number of SIRS criteria and higher APPLE scores (Table 1) all suggest that the septic shock population enrolled was more severely affected than the cats with sepsis alone.

Individually, the concentrations of Flt-3L, IL-12 and MCP-1 were moderately discriminant for septic shock in the present population. In a multivariable model, the combination of these three cytokines differentiated sepsis from septic shock in over 91% of cases. These three cytokines have distinct but linked physiologic roles. MCP-1 is a member of the chemotactic cytokine family that functions to recruit monocytes and modulate T-cell function (44); Flt-3L causes dendritic cell and lymphocyte proliferation (45); IL-12 is a predominantly pro-inflammatory cytokine that promotes T-helper cell development and activity (46). These latter two cytokines may be linked because Flt-3L can increase the number of dendritic cells in septic shock thereby increasing IL-12 concentrations (47). It is not clear why these cytokines differentiate sepsis severity in cats, and further investigation appears warranted, but MCP-1 has been documented to be increased (12), and to have prognostic value in dogs with sepsis (13).

Interestingly, >50% of cats had one or more measured concentration that was lower than the LLD for 15 of the 19 cytokines for healthy controls, and for 11 cytokines for cats with sepsis and septic shock. This inevitably limited our evaluation of the utility of these markers and may warrant reassessment of the utility of the full 19-plex assay in cats. As the present study involved an exploratory analysis of cytokines in feline sepsis it was desirable to measure the maximum number of cytokines, similar to other exploratory studies in related fields (20, 48). Additionally, there was no clear difference between healthy controls and sick cats (sepsis and septic shock) for the majority of the investigated analytes. The lack of difference between healthy and sick cats could be partially due to the low analytical sensitivity of the assay used, as previously reported (21, 22). This may be a limitation of the assay technology since similar limitations with undetectable analytes has been widely reported in canine studies (49). We speculate that this may result from difficulties designing an assay that can measure cytokine concentrations that can vary by multiple orders of magnitude even within healthy canine populations (11, 50). The potential impact of sample handling and storage should also be considered since some of the samples were stored for extended periods. Unpredictable pathophysiological differences including activation (or inactivation) of specific immune cell lines and immune paralysis may be the reasons for the low circulating concentrations of many cytokines/chemokines in our study population. Leukocyte anergy and hyporeactivity are well-described phenomena in spontaneous and experimental sepsis, and are associated with reduced production of cytokines and inflammatory mediators from affected leukocytes (26). Finally, some cytokines show low circulating concentrations in the face of increased local tissue activity, according to the concept of compartmentalization of sepsis. The effect of this compartmentalization is to reduce the concentrations of cytokines in circulation which in turn may not correlate with their local concentrations or functional activities in target tissues (19, 26, 49).

This study has some limitations that should be acknowledged. The sample size was large for feline sepsis studies, but remains relatively small and was heterogeneous in terms of sepsis etiologies. Although euthanasia was performed only after clinical perception of moribund condition or end-stage disease (cases euthanized for financial reasons were excluded) the potential impact of euthanasia should be considered a possible source of bias, particularly with regards to the outcome analyses. Additionally, although the sampling was performed prospectively using standardized procedures for sample collection and storage, minor variations in sample processing (e.g., delay in cryopreservation) could have negatively affected analytes concentrations. The intrinsic limitations of the multiplex assay should be considered, as it is conceivable that concentrations of other analytes could have been significantly different between groups, but remained undetected because of the limited assay sensitivity. In this regard, the substitution of out-of-range concentrations with the respective LLD reported by the assay could have biased statistical outcomes (49, 51). Similarly, cytokines/chemokines results were reported as observed concentrations, and not as MFI. Some authors recommend using fluorescence intensity results for multiplexed cytokine assays to increase statistical power and eliminate standard curve range and linearity issues (25). However, MFI values are only useful if machine calibration is totally consistent between runs, or when a single assay contains all the measured samples (49). Due to the triplicate measurements and the number of samples involved in the present study, multiple assays performed over several days were necessary, which precluded use of MFI values.

To conclude, this is the first study describing the cytokine and chemokine network in cats with both sepsis and septic shock. The cytokines IL-6, IL-8, KC-like, and RANTES appear to be important for the pathogenesis of both sepsis and septic shock in cats, but these biomarkers are not discriminating for sepsis severity or outcome. Concentrations of the other cytokines investigated suggest they may not be useful for the assessment or investigation of sepsis in cats. Immune paralysis and leukocyte hyporeactivity could have been responsible for the low concentrations of these inflammatory mediators in our study population, but assay-related problems cannot be ruled out. Further prospective studies are needed to evaluate cytokine profiling in specific subsets of feline sepsis, and could contribute to immunophenotyping of septic individuals according to sepsis severity and underlying disease.

## Data Availability Statement

All datasets generated for this study are included in the article/supplementary material.

## Ethics Statement

All samples analyzed in this study were collected from cats managed at the two participating veterinary teaching hospitals (Cornell University, Ithaca, NY, USA and University of Bologna, Italy) as part of studies approved by the local Institutional Animal Care and Use Committees (IACUC), and undertaken under written informed client consent (Cornell IACUC 2014-0053; Bologna DL 26/2014, Project 581). Healthy privately owned cats were enrolled as controls with local Institutional Animal Care and Use Committees approval and written informed client consent (Cornell IACUC 2014-0052).

## Author Contributions

RT assisted with the study design, collected and analyzed the data, and co-wrote the manuscript. GM, CA, DL-P, and MG collected and analyzed the data, and edited the manuscript. RG designed the study, collected and analyzed the data, and co-wrote the manuscript. All authors contributed to read and approved the final manuscript.

## Conflict of Interest

The authors declare that the research was conducted in the absence of any commercial or financial relationships that could be construed as a potential conflict of interest.

## Abbreviations

A/G: albumin to globulin ratio
ALP: alkaline phosphatase
ALT: alanine transaminase
APPLE: acute patient physiologic and laboratory evaluation
AST: aspartate transaminase
BE: base excess
CCL-2: C-C motif chemokine ligand-2
Flt-3L: FMS-like tyrosine kinase-3 ligand
GGT: ɤ-glutamyltransferase
GM-CSF: granulocyte macrophage-colony stimulating factor
Hb: hemoglobin concentration
HCT: hematocrit value
ICU: intensive care unit
IFN-γ: interferon-γ
IL: interleukin
KC-like: keratinocyte chemoattractant-like
LLD: lower limit of detection
MCHC: mean cell hemoglobin concentration
MCP-1: monocyte chemoattractant protein-1
MCV: mean corpuscular volume
MFI: median fluorescence intensity
PDGF-BB: platelet-derived growth factor-BB
RANTES: Regulated upon Activation, Normal T-cell Expressed and Secreted
RDW: red cell distribution width
RI: reference interval
SAA: serum-amyloid A
SBP: systolic blood pressure
SCF: stem cell factor
SDF-1: stromal cell-derived factor 1
SIRS: systemic inflammatory response syndrome
TNF-α: tumor-necrosis factor-α
WBC: white blood cell.

## References

[1] Wiersinga WJ Leopold SJ Cranendonk DR van der Poll T. Host innate immune responses to sepsis. Virulence. (2014) 5:36–44. 10.4161/viru.25436 23774844 PMC3916381

[2] Chaudhry H Zhou J Zhong Y Ali MM McGuire F Nagarkatti PS. Role of cytokines as a double-edged sword in sepsis. In vivo. (2013) 27:669–84. 24292568 PMC4378830

[3] Jacobi J. Pathophysiology of sepsis. Am J Health Syst Pharm. (2002) 59:S3–8. 10.1093/ajhp/59.suppl_1.S3 11885412

[4] Glauser MP. Pathophysiologic basis of sepsis: considerations for future strategies of intervention. Crit Care Med. (2000) 28:S4–8. 10.1097/00003246-200009001-00002 11007189

[5] Levy MM Fink MP Marshall JC Abraham E Angus D Cook D. SCCM/ESICM/ACCP/ATS/SIS International sepsis definitions conference. Crit Care Med. (2003) 31:1250–6. 10.1097/01.CCM.0000050454.01978.3B 12682500

[6] Shankar-Hari M Phillips GS Levy ML Seymour CW Liu VX Deutschman CS. Developing a new definition and assessing new clinical criteria for septic shock: for the third international consensus definitions for sepsis and septic shock (Sepsis-3). JAMA. (2016) 315:775–87. 10.1001/jama.2016.0289 26903336 PMC4910392

[7] Sriskandan S Altmann DM. The immunology of sepsis. J Pathol. (2008) 214:211–23. 10.1002/path.2274 18161754

[8] Peters van Ton AM Kox M Abdo WF Pickkers P. Precision immunotherapy for sepsis. Front Immunol. (2018) 9:1926. 10.3389/fimmu.2018.01926 30233566 PMC6133985

[9] Pickkers P Kox M. Towards precision medicine for sepsis patients. Crit Care. (2017) 21:11. 10.1186/s13054-016-1583-z 28077168 PMC5228110

[10] Karlsson I Hagman R Johannisson A Wang L Sodersten F Wernersson S. Multiplex cytokine analyses in dogs with pyometra suggest involvement of KC-like chemokine in canine bacterial sepsis. Vet Immunol Immunopathol. (2016) 170:41–6. 10.1016/j.vetimm.2016.01.005 26837616

[11] Johnson V Burgess B Morley P Bragg R Avery A Dow S. Comparison of cytokine responses between dogs with sepsis and dogs with immune-mediated hemolytic anemia. Vet Immunol Immunopathol. (2016) 180:15–20. 10.1016/j.vetimm.2016.08.010 27692090

[12] Martiny P Goggs R. Biomarker guided diagnosis of septic peritonitis in dogs. Front Vet Sci. (2019) 6:208. 10.3389/fvets.2019.00208 31316998 PMC6610427

[13] Goggs R Letendre JA. Evaluation of the host cytokine response in dogs with sepsis and noninfectious systemic inflammatory response syndrome. J Vet Emerg Crit Care. (2019) 29:593–603. 10.1111/vec.12903 31637812

[14] Brady CA Otto CM van Winkle TJ King LG. Severe sepsis in cats: 29 cases (1986-1998). J Am Vet Med Assoc. (2000) 217:531–5. 10.2460/javma.2000.217.531 10953718

[15] Babyak JM Sharp CR. Epidemiology of systemic inflammatory response syndrome and sepsis in cats hospitalized in a veterinary teaching hospital. J Am Vet Med Assoc. (2016) 249:65–71. 10.2460/javma.249.1.65 27308883

[16] Paltrinieri S. The feline acute phase reaction. Vet J. (2008) 177:26–35. 10.1016/j.tvjl.2007.06.005 17686640 PMC7128355

[17] Troia R Gruarin M Foglia A Agnoli C Dondi F Giunti M. Serum amyloid A in the diagnosis of feline sepsis. J Vet Diagn Invest. (2017) 29:856–9. 10.1177/1040638717722815 28754082

[18] Declue AE Delgado C Chang CH Sharp CR. Clinical and immunologic assessment of sepsis and the systemic inflammatory response syndrome in cats. J Am Vet Med Assoc. (2011) 238:890–7. 10.2460/javma.238.7.890 21453177

[19] Gruen ME Messenger KM Thomson AE Griffith EH Paradise H Vaden S. A comparison of serum and plasma cytokine values using a multiplexed assay in cats. Vet Immunol Immunopathol. (2016) 182:69–73. 10.1016/j.vetimm.2016.10.003 27863553 PMC5522725

[20] Gruen ME Messenger KM Thomson AE Griffith EH Aldrich LA Vaden S. Evaluation of serum cytokines in cats with and without degenerative joint disease and associated pain. Vet Immunol Immunopathol. (2017) 183:49–59. 10.1016/j.vetimm.2016.12.007 28063477 PMC5522727

[21] Halpin RE Saunders RS Thompson BJ Rohde Newgent AS Amorim J Melillo GN. Evaluation of a feline-specific multiplex, bead-based assay for detection of cytokines, chemokines, growth factors, and other immunologically active proteins in serum and plasma samples from cats. Am J Vet Res. (2016) 77:495–504. 10.2460/ajvr.77.5.495 27111017

[22] Parys M Yuzbasiyan-Gurkan V Kruger JM. Serum cytokine profiling in cats with acute idiopathic cystitis. J Vet Intern Med. (2018) 32:274–9. 10.1111/jvim.15032 29356123 PMC5787166

[23] Singer M Deutschman CS Seymour CW Shankar-Hari M Annane D Bauer M. The third international consensus definitions for sepsis and septic shock (Sepsis-3). JAMA. (2016) 315:801–10. 10.1001/jama.2016.0287 26903338 PMC4968574

[24] Hayes G Mathews K Doig G Kruth S Boston S Nykamp S. The feline acute patient physiologic and laboratory evaluation (Feline APPLE) score: a severity of illness stratification system for hospitalized cats. J Vet Intern Med. (2011) 25:26–38. 10.1111/j.1939-1676.2010.0648.x 21143303

[25] Breen EJ Polaskova V Khan A. Bead-based multiplex immuno-assays for cytokines, chemokines, growth factors and other analytes: median fluorescence intensities versus their derived absolute concentration values for statistical analysis. Cytokine. (2015) 71:188–98. 10.1016/j.cyto.2014.10.030 25461398

[26] Cavaillon JM Adib-Conquy M Fitting C Adrie C Payen D. Cytokine cascade in sepsis. Scand J Infect Dis. (2003) 35:535–44. 10.1080/00365540310015935 14620132

[27] Harbarth S Holeckova K Froidevaux C Pittet D Ricou B Grau GE. Diagnostic value of procalcitonin, interleukin-6, and interleukin-8 in critically ill patients admitted with suspected sepsis. Am J Respir Crit Care Med. (2001) 164:396–402. 10.1164/ajrccm.164.3.2009052 11500339

[28] Qiu X Zhang L Tong Y Qu Y Wang H Mu D. Interleukin-6 for early diagnosis of neonatal sepsis with premature rupture of the membranes: a meta-analysis. Medicine (Baltimore). (2018) 97:e13146. 10.1097/MD.0000000000013146 30461611 PMC6392693

[29] Henning DJ Hall MK Watsjold BK Bhatraju PK Kosamo S Shapiro NI. Interleukin-6 improves infection identification when added to physician judgment during evaluation of potentially septic patients. Am J Emerg Med. (2019). 10.1016/j.ajem.2019.158361. [Epub ahead of Print]. 31375355

[30] Shao WX Yu DJ Zhang WY Wang XJ. Clinical significance of interleukin-6 in the diagnosis of sepsis and discriminating sepsis induced by gram-negative bacteria. Pediatr Infect Dis J. (2018) 37:801–5. 10.1097/INF.0000000000001904 30004393

[31] Rau S Kohn B Richter C Fenske N Kuchenhoff H Hartmann K. Plasma interleukin-6 response is predictive for severity and mortality in canine systemic inflammatory response syndrome and sepsis. Vet Clin Pathol. (2007) 36:253–60. 10.1111/j.1939-165X.2007.tb00220.x 17806073

[32] Palmiere C Augsburger M. Markers for sepsis diagnosis in the forensic setting: state of the art. Croat Med J. (2014) 55:103–14. 10.3325/cmj.2014.55.103 24778096 PMC4009711

[33] Haas M Kaup FJ Neumann S. Canine pyometra: a model for the analysis of serum CXCL8 in inflammation. J Vet Med Sci. (2016) 78:375–81. 10.1292/jvms.15-0415 26522810 PMC4829503

[34] Kostic I Gurrieri C Piva E Semenzato G Plebani M Caputo I. Comparison of presepsin, procalcitonin, interleukin-8 and C-reactive protein in predicting bacteraemia in febrile neutropenic adult patients with haematological malignancies. Mediterr J Hematol Infect Dis. (2019) 11:e2019047. 10.4084/mjhid.2019.047 31528313 PMC6736337

[35] Murakami K Maeda S Yonezawa T Matsuki N. CC chemokine ligand 2 and CXC chemokine ligand 8 as neutrophil chemoattractant factors in canine idiopathic polyarthritis. Vet Immunol Immunopathol. (2016) 182:52–8. 10.1016/j.vetimm.2016.09.009 27863550

[36] Goggs R Letendre JA. High mobility group box-1 and pro-inflammatory cytokines are increased in dogs after trauma but do not predict survival. Front Vet Sci. (2018) 5:179. 10.3389/fvets.2018.00179 30105229 PMC6077187

[37] Galan A Mayer I Rafaj RB Bendelja K Susic V Ceron JJ. MCP-1, KC-like and IL-8 as critical mediators of pathogenesis caused by Babesia canis. PLoS ONE. (2018) 13:e0190474. 10.1371/journal.pone.0190474 29304171 PMC5756041

[38] Karlsson I Hagman R Guo Y Humblot P Wang L Wernersson S. Pathogenic escherichia coli and lipopolysaccharide enhance the expression of IL-8, CXCL5, and CXCL10 in canine endometrial stromal cells. Theriogenology. (2015) 84:34–42. 10.1016/j.theriogenology.2015.02.008 25765298

[39] Kaukonen KM Bailey M Pilcher D Cooper DJ Bellomo R. Systemic inflammatory response syndrome criteria in defining severe sepsis. N Engl J Med. (2015) 372:1629–38. 10.1056/NEJMoa1415236 25776936

[40] Churpek MM Zadravecz FJ Winslow C Howell MD Edelson DP. Incidence and prognostic value of the systemic inflammatory response syndrome and organ dysfunctions in ward patients. Am J Respir Crit Care Med. (2015) 192:958–64. 10.1164/rccm.201502-0275OC 26158402 PMC4642209

[41] Sharp CR Systemic inflammatory response syndrome, sepsis, and multiple organ dysfunction syndrome. In: Drobatz KJ Hopper K Rozanski E Silverstein DC editors. Textbook of Small Animal Emergency Medicine. 2. 1st ed Hoboken, NJ: Wiley Blackwell (2019). p. 1030–7. 10.1002/9781119028994.ch159

[42] Okano S Yoshida M Fukushima U Higuchi S Takase K Hagio M. Usefulness of systemic inflammatory response syndrome criteria as an index for prognosis judgement. Vet Rec. (2002) 150:245–6. 10.1136/vr.150.8.245 11916026

[43] Rosenstein PG Tennent-Brown BS Hughes D. Clinical use of plasma lactate concentration. Part 1: physiology, pathophysiology, and measurement. J Vet Emerg Crit Care. (2018) 28:85–105. 10.1111/vec.12708 29533512

[44] Deshmane SL Kremlev S Amini S Sawaya BE. Monocyte chemoattractant protein-1 (MCP-1): an overview. J Interferon Cytokine Res. (2009) 29:313–26. 10.1089/jir.2008.0027 19441883 PMC2755091

[45] Liu K Nussenzweig MC. Origin and development of dendritic cells. Immunol Rev. (2010) 234:45–54. 10.1111/j.0105-2896.2009.00879.x 20193011

[46] Vignali DA Kuchroo VK. IL-12 family cytokines: immunological playmakers. Nat Immunol. (2012) 13:722–8. 10.1038/ni.2366 22814351 PMC4158817

[47] Wysocka M Montaner LJ Karp CL. Flt3 ligand treatment reverses endotoxin tolerance-related immunoparalysis. J Immunol. (2005) 174:7398–402. 10.4049/jimmunol.174.11.7398 15905588

[48] O'Halloran C McCulloch L Rentoul L Alexander J Hope JC Gunn-Moore DA. Cytokine and chemokine concentrations as biomarkers of feline mycobacteriosis. Sci Rep. (2018) 8:17314. 10.1038/s41598-018-35571-5 30470763 PMC6251861

[49] Richter KR Nasr AN Mexas AM. Cytokine concentrations measured by multiplex assays in canine peripheral blood samples. Vet Pathol. (2018) 55:53–67. 10.1177/0300985817725388 28812527

[50] Nikolic Nielsen L Kjelgaard-Hansen M Kristensen AT. Monocyte chemotactic protein-1 and other inflammatory parameters in Bernese mountain dogs with disseminated histiocytic sarcoma. Vet J. (2013) 198:424–8. 10.1016/j.tvjl.2013.07.030 23992872

[51] Helsel DR. Fabricating data: how substituting values for nondetects can ruin results, and what can be done about it. Chemosphere. (2006) 65:2434–9. 10.1016/j.chemosphere.2006.04.051 16737727

